# 3D Molecular Cytology of Hop (*Humulus lupulus*) Meiotic Chromosomes Reveals Non-disomic Pairing and Segregation, Aneuploidy, and Genomic Structural Variation

**DOI:** 10.3389/fpls.2018.01501

**Published:** 2018-11-01

**Authors:** Katherine A. Easterling, Nicholi J. Pitra, Rachel J. Jones, Lauren G. Lopes, Jenna R. Aquino, Dong Zhang, Paul D. Matthews, Hank W. Bass

**Affiliations:** ^1^Department of Biological Science, Florida State University, Tallahassee, FL, United States; ^2^Hopsteiner, S.S. Steiner, Inc., New York, NY, United States

**Keywords:** cytogenetics, FISH, non-Mendelian inheritance, segregation distortion, telomere bouquet

## Abstract

Hop (*Humulus lupulus* L.) is an important crop worldwide, known as the main flavoring ingredient in beer. The diversifying brewing industry demands variation in flavors, superior process properties, and sustainable agronomics, which are the focus of advanced molecular breeding efforts in hops. Hop breeders have been limited in their ability to create strains with desirable traits, however, because of the unusual and unpredictable inheritance patterns and associated non-Mendelian genetic marker segregation. Cytogenetic analysis of meiotic chromosome behavior has also revealed conspicuous and prevalent occurrences of multiple, atypical, non-disomic chromosome complexes, including those involving autosomes in late prophase. To explore the role of meiosis in segregation distortion, we undertook 3D cytogenetic analysis of hop pollen mother cells stained with DAPI and FISH. We used telomere FISH to demonstrate that hop exhibits a normal telomere clustering bouquet. We also identified and characterized a new sub-terminal 180 bp satellite DNA tandem repeat family called HSR0, located proximal to telomeres. Highly variable 5S rDNA FISH patterns within and between plants, together with the detection of anaphase chromosome bridges, reflect extensive departures from normal disomic signal composition and distribution. Subsequent FACS analysis revealed variable DNA content in a cultivated pedigree. Together, these findings implicate multiple phenomena, including aneuploidy, segmental aneuploidy, or chromosome rearrangements, as contributing factors to segregation distortion in hop.

## Introduction

*Humulus lupulus* (hop), a member of the Cannabaceae family of flowering plants, is a dioecious, high-climbing, herbaceous bine that is best known as a flavoring agent in beer. Hop cultivation for this purpose has been traced back to Germany, 736 AD (Neve, 1991). For hop, despite its long successful history of domestication, modern breeding practices are associated with a number of challenges. For instance, although hop is generally cultivated vegetatively by rhizomes, sexual crosses are necessary in order to breed for new disease-resistant and chemically desirable varieties. The long history of cultivation includes colchicine-induced polyploidization (Roborgh, 1969) and the introgression of genetically distinct wild populations (Small, 1980; Reeves and Richards, 2011). Recent genetic, genomic, and quantitative trait analyses have demonstrated that the genome of hop is complex and structurally diverse (Zhang et al., 2017). Irregularities in hop transmission genetics are reflected in non-Mendelian segregation distortion and sex ratio bias (Seefelder et al., 2000; Jakse et al., 2008, 2011; McAdam et al., 2013, 2014).

Meiotic chromosome behavior is implicated as a cause for the unusual transmission genetics that are found in hop (Zhang et al., 2017). In sexually reproducing organisms, meiosis reduces the genomes from diploid to haploid (John, 1990) and homologous chromosomes from each parental genome undergo pairing, synapsis, recombination (John, 1990). These events ensure proper segregation of chromosomes into balanced gametes for subsequent fertilization and transmission to the progeny of the next generation (Stebbins, 1935; John, 1990; Murphy and Bass, 2012). Deviations from such normal disomic pairing and disjunction can lead to a variety of genetic inheritance problems including gene dosage imbalance, aneuploidy, and chromosomal rearrangements.

Among the hallmarks of meiosis that are cytologically evident are (1) the presence of a telomere bouquet in early prophase that guides homology search and subsequent synapsis, (2) the presence of bivalents at diakinesis during late prophase, (3) complete and equal separation of homologs at meiosis I, and (4) the production of 4 haploid nuclei with 1:1:1:1 segregation, as evidenced from tetrad analysis. For hop, non-disomic and heteromorphic sex chromosome figures have been noted and speculated to impact segregation patterns (Sinotô, 1929; Jacobsen, 1957; Neve, 1958; Haunold, 1991; Shephard et al., 2000; Zhang et al., 2017). However, neither the early prophase telomere bouquet nor the post-meiotic segregation has been characterized cytogenetically.

Here we set out to further examine the cytogenetics of male hop meiosis. To date, several somatic karyotypes for hop have been produced (Sinotô, 1929; Winge, 1929; Shephard et al., 2000). The more recent hop karyotypes include FISH-mapped loci for the *Humulus lupulus*-specific subtelomeric repeat-1 (HSR1), the Humulus japonicus-specific subtelomeric repeat (HSJR), the nucleolus organizer region (NOR), and 5S rDNA (Karlov et al., 2003; Divashuk et al., 2011; Alexandrov et al., 2012). Telomere FISH has also been used in hop, but not to test for the presence of the telomere bouquet at early prophase. In this study, we employed 3D acrylamide FISH (Howe et al., 2013) to show that hop has a classical zygotene telomere bouquet, but non-uniform segregation of 5S rDNA loci. Detection of anaphase bridges and variable DNA content further implicate genome structure variability in the unusual inheritance patterns of hop.

## Methods

### Plant materials and fixation

Plants were collected and fixed according to previously described methods (Zhang et al., 2017), with exceptions on timing and paraformaldehyde concentration during the meiocyte buffer A (MBA) steps. Briefly, hop panicles were field collected and immediately fixed in Farmer's fluid (3:1 ethanol:acetic acid) overnight, replaced with Farmer's fluid for a second overnight period, and exchanged into 70% ethanol for storage at −20C. Hopsteiner varieties were collected on site in the company's male yard, and were grown under standard agronomic conditions at the Golden Gate Ranches, S.S. Steiner, Inc, Yakima, WA. The *H. lupulus* var. *neomexicanus*, plant SH2, was collected in the Coronado National Forest in Arizona.

### Bioinformatic identification of tandem repeat family, *H. lupulus* subterminal repeat 0, HSR0

DNA from Apollo was used to make a library of large DNA fragments using the RSII technology. PacBio Single Molecule, Real-Time (SMRT) DNA sequences were manually and randomly screened for tandem repeats using the dot plot function of GenomeMatcher (Ohtsubo et al., 2008). Tandem repeats in the clones were detected as parallel stripes exhibiting a striping pattern evenly spaced and parallel to the main diagonal of identity. This striping pattern is diagnostic for DNA containing tandemly repeated sequences, easily detected by dot plot inspection, and was used to find new candidate loci for FISH probe development. Potential hits were further analyzed by dot-plotting against other hits to detect “allelism” among the repeats found within individual clones. Computational tandem repeat searching was done using “ksift” (https://github.com/dvera/ksift) Jellyfish-2 (https://github.com/zippav/Jellyfish-2) and YASS dot-plotter (https://github.com/laurentnoe/yass).

The two most frequent tandem repeat families found in this way were the ~385 bp tandemly repeated sequence previously defined as the *Humulus*-specific subtelomeric repeat-1, HSR1, GenBank Accession GU831574, (Divashuk et al., 2014), and a new ~180 bp tandemly-repeated sequence designated here as *H. lupulus* Subterminal Repeat 0, HSR0. The names of five HSR0-containing Apollo genomic DNA sequences from PacBio SMRT and their length and GenBank Accession numbers are HuluTR180-120 (6,005 bp, Acc. MH188533), HuluTR180-69 (11,761 bp, Acc. MH188534), HuluTR180TEL-954 (10,443 bp, Acc. MH188536), HuluTR180TEL-316 (5,717 bp, Acc. MH188537), and HuluTR180-270 (10,794 bp, Acc. MH188538). Two of these, HuluTR180TEL-316 and HuluTR180TEL-316, contain both HSR0 and telomeric repeats. For comparison, a clone with telomeric DNA repeats but lacking HSR0 has also been deposited as HuluTEL-347 (5,551 bp, Acc. MH188535).

### Fluorescence *in-situ* hybridization, FISH

For 3D FISH, whole Farmer's-fixed buds attached to peduncles were equilibrated in meiocyte Buffer A (Bass et al., 1997) for 30 min at RT, repeated twice, followed by fixation in 1% formaldehyde in MBA for 1 h at RT. After fixation, the tissue was washed three times in MBA for 15-min each at RT and stored in MBA at 4C. Meiotic cells were micro-dissected from buds of various sizes spanning meiosis (from ~1.5 to 2.5 mm in length) and embedded in acrylamide as described for the 3D acrylamide FISH technique (Howe et al., 2013).

FISH probes used in this study were synthetic oligonucleotides co-synthetically coupled to fluorescent dyes and designed to detect telomeres (“MTLF-29,” 5′-[FITC]-(CCCTAAA)_4_-3′ or “MTLY-28-16” 5′-[ATTO647N]-(CCCTAAA)_4_-3′), HSR0 tandem repeats (“ZERO-Y,” 5′-[ATTO647N]-AGAAATATGAGTGAATTACGAAATCGC-3′), HSR1 tandem repeats (“HSR1a_22,” 5′-Alexa488-GGTACCCCTCTGGTGAATTGGA-3′), or 5S rDNA (pool of three oligos: “5SBOB1F,” 5′-[Alexa488]-GCACCGGATCCCATCAGAACTCC-3′]; “5SBOB2F,” 5′-[Alexa488]-AGTTAAGCGTGCTTGGGCGAGAG-3′; and “5SBOB3F,” 5′-[Alexa488]-GTGACCTCCTGGGAAGTCCTCGTG-3′). The three 5S rDNA probes were selected on the basis of their 100% identity, according to the 5S rRNA Database (Szymanski et al., 2002), for *Cannabis sativa* (5sRNAdb Record ID E00464), *Gossypium hirsutum* (5sRNAdb Record ID E00193), and *Zea mays* (5sRNAdb Record ID E00011). Prehybridization, hybridization, post-hybridization washes, DAPI counterstaining, and slide mounting were done as described (Howe et al., 2013) using denaturation temperatures ranging from 88 to 92°C.

### Collection, analysis, and display of 3D deconvolution microscopy images

Three-dimensional images were collected on a DeltaVision deconvolution microscope, using a 60X lens and 0.2 micron Z-step optical sections as described (Zhang et al., 2017) for DAPI imaging, but also including FITC, TRITC, or CY5 imaging for FISH probes that fluoresce green, red, or far-red, respectively. Distance measurements between the 5S rDNA FISH signals were obtained as point-to-point Euclidean distances using the *Measure Distances* program, manually selecting the brightest voxel centered in the X, Y, and Z dimensions for any given FISH signal.

### FACS analysis of DNA content

A two-step protocol (Pellicer and Leitch, 2014) was followed to isolate hop nuclei from 20 mg of fresh leaf tissue chopped and stained in LB01 buffer. The homogenate was filtered through a 30–42 um nylon mesh filter, centrifuged, and resuspended in LB01 buffer. Samples were vortexed before being analyzed at the Iowa State University (Ames, IA) Flow Cytometry Facility using an unmodified BD Biosciences FACSCanto (San Jose, CA) flow cytometer.

## Results

### Telomere FISH reveals bouquet formation in hop

**Figure 1:**
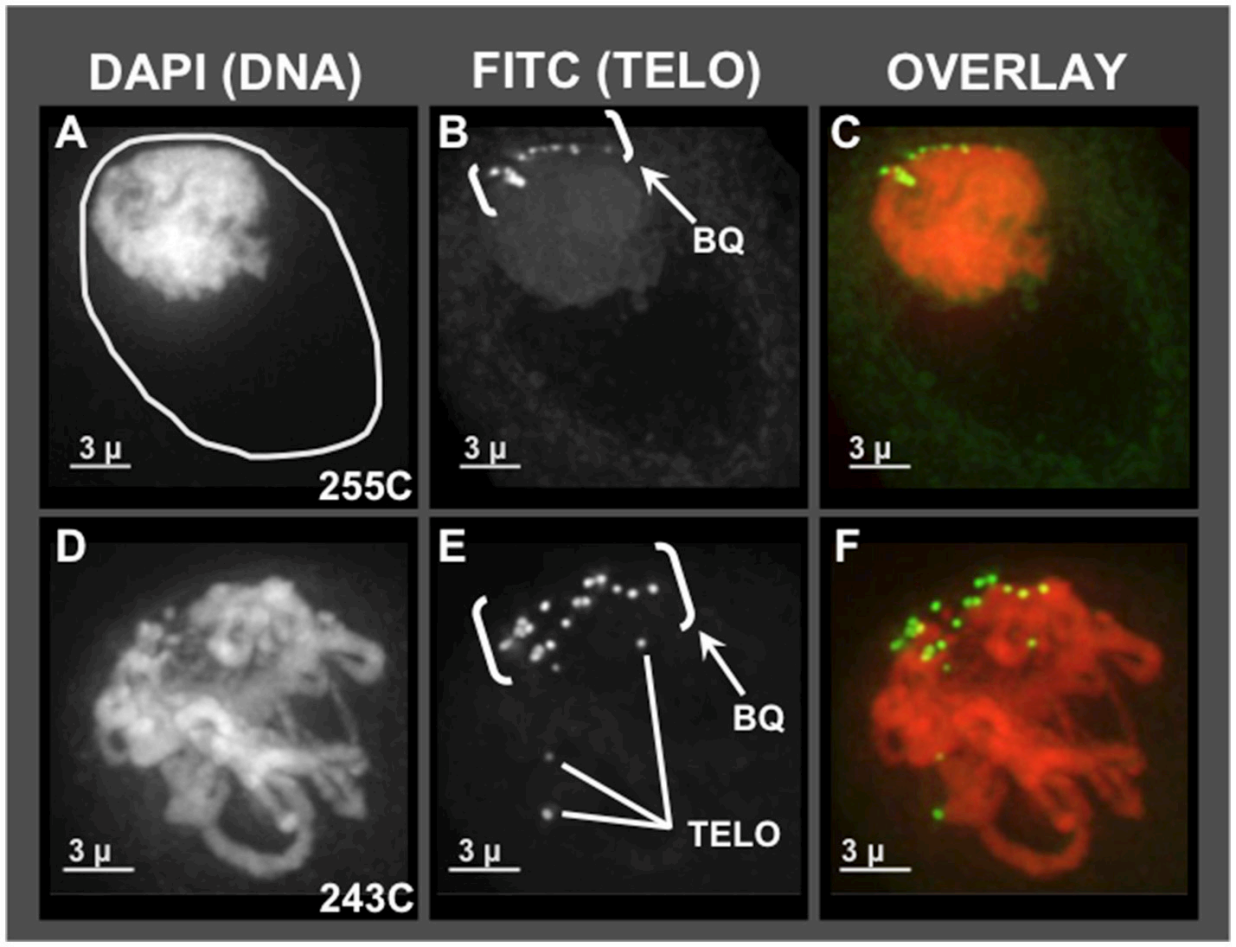
Telomere bouquet in pollen mother cells of hop. Male flower buds were harvested and fixed in Farmer's Fluid, then exchanged into Buffer A and formaldehyde fixed before microdissecting pollen mother cells from anthers for 3D acrylamide FISH. Through-focus projections of nuclei, one nucleus per row, are shown as through-focus maximum-intensity projections in gray-scale for individual wavelengths or in color for overlay images, as labeled across the top. **(A–C)** Hop nucleus from plant 255C at early prophase showing the nuclear periphery (**A**- white circle) and the telomere cluster region (bracketed, BQ). **(D–F)** Hop nucleus from plant 243C at early-middle prophase showing the bouquet (BQ), with three examples of single telomere FISH signals (Tel, **E**) indicated.

Given the pervasive irregularities noted in Zhang et al. (2017) for meiotic chromosome behavior together with SD, we first wanted to ask which, if any, of the cytological hallmarks of normal meiotic prophase are found in hop using molecular cytology. The unusual chromosome interactions and genetic segregation defects previously observed in hop could result from missing or faulty bouquet, given the importance of that structure in efficient pairing and recombination. For comparison, a normal bouquet involves the clustering of telomeres on the nuclear envelope in early meiotic prophase. Specifically, the bouquet stage actually spans from late leptotene, through all of zygotene, and into early pachytene (Bass et al., 1997; Bass, 2003; Scherthan, 2007). Using 3D telomere FISH, we document the presence of a telomere bouquet, as summarized in Figure 1. Of the hop nuclei exhibiting a telomere FISH bouquet (*n* = 59 from plants 255C, 243C, 243D, Male 15), 36 nuclei showed tightly clustered signals and 23 nuclei showed less tightly clustered FISH signals at the nuclear periphery. For two representative hop nuclei at early meiotic prophase, the telomere FISH signals are clustered in one area of the nuclear periphery, forming a normal-looking bouquet (Figures 1B,E, labeled “BQ”) (Bass et al., 1997). The first nucleus (Figures 1A–C) shows a common pattern in which the nuclear volume (Figure 1A, circle traces at the nucleus-cytoplasm boundary) is larger than that occupied by the chromatin mass. This clumping of chromatin is a zygotene stage-specific, coagulative-fix-dependent artifact, producing the so-called the synizetic knot, as described for *Oenothera* and other plant species (Golczyk et al., 2008). A second nucleus (Figures 1D–F) shows that the bouquet is clearly present in a nucleus that appears to be a later stage than the first nucleus, but still in early prophase, as evidenced by the presence of discrete chromosome fibers. These experiments showed that the telomere bouquet does indeed occur in hop male meiocytes, and was observed in both modern cultivars (Figure 1) and wild-collected plants (not shown). From these analyses, we find no compelling evidence to implicate a faulty or missing bouquet as causal for segregation distortion in hop. These analyses thereby establish that the bouquet appears normal in both structure and timing.

### HSR0 is an abundant subtelomeric 180 bp satellite DNA tandem repeat

In order to further characterize the behavior of hop meiotic chromosomes, we set out develop new FISH probes for cytological tracking of multiple and specific regions of chromosomes. New probes would help solve the problem that hop chromosomes are very similar in centromere location and size but linkage groups remain largely unassigned to chromosomes. We focused on tandem repeats as a class of sequences that make for ideal FISH probes suitable for karyotype development (Albert et al., 2010; Divashuk et al., 2011) They yield bright, discrete signals that can be seen to pair and segregate as reporter loci in meiosis (Bass et al., 2000, 2003).

**Figure 2:**
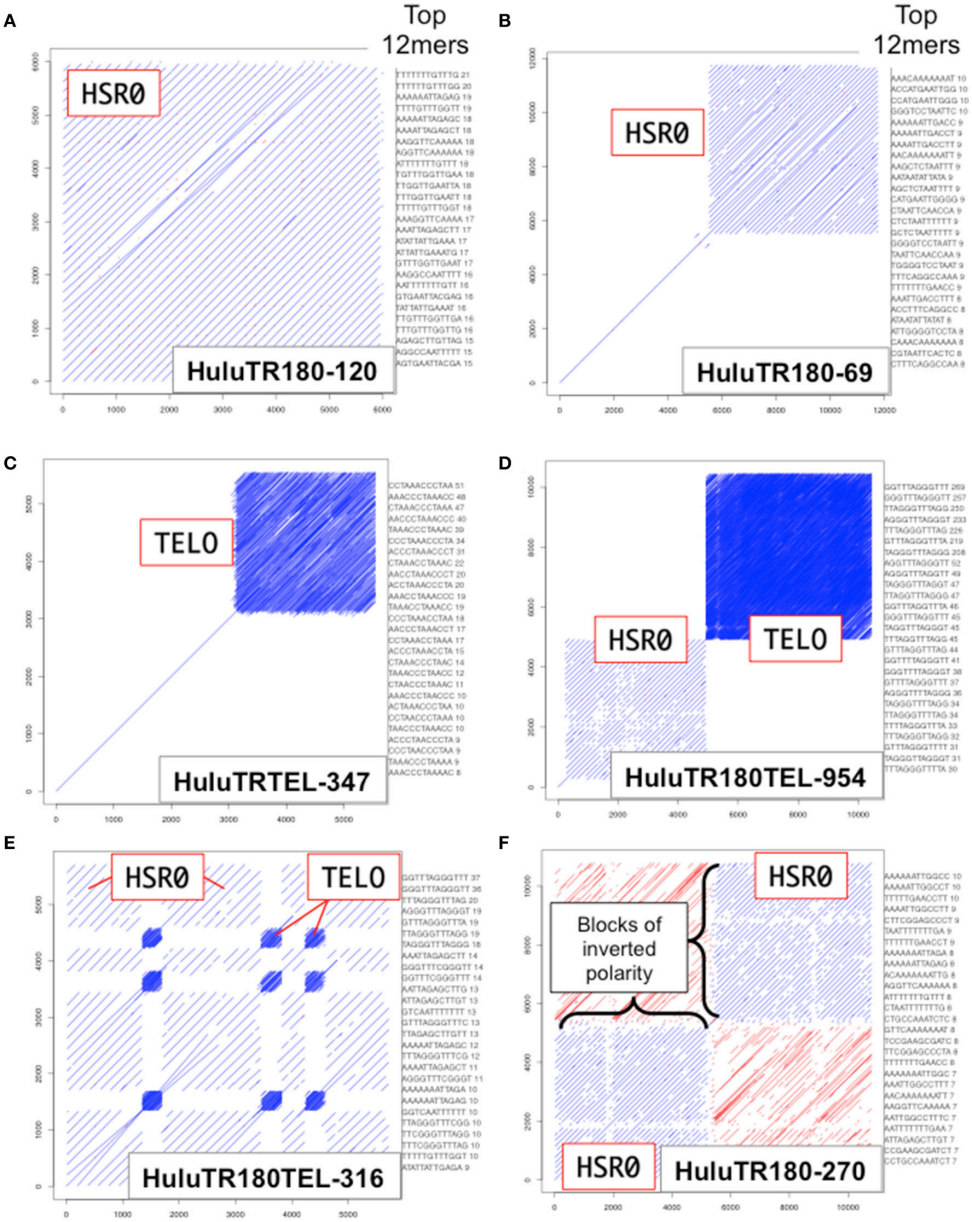
HSR0 dot plot outputs of tandemly repetitive genomic regions. PacBio Single Molecule, Real-Time (SMRT) DNA sequences were screened for tandem repeats (Ohtsubo et al., 2008). For each clone, a self dot-plot is shown along with the top 20 12mers for that clone using kmer analysis. The stripes represent internal tandem repeats of HSR0 (blue stripes) or telomere repeats (tightly packed blue stripe blocks). **(A)** The HuluTR180-120 clone showing HSR0 repeat occupies the entire 6 Kbp clone. **(B)** The HuluTR180-69 clone showing HSR0 repeat occupies around one half of the 6 Kbp clone, whereas the single diagonal represents sequences that are not repeated within the clone. **(C)** The HuluTRTEL-347 clone showing with unique sequences in the first 3 kbp followed by telomere repeat DNA (CCCTAAA_n_) in the last ~3 kbp. **(D)** The HuluTR180TEL-954 clone showing a large block of HSR0 repeats immediately followed by more than 5 Kbp of telomere repeat DNA (TTTAGGG_n_). **(E)** The HuluTR180TEL-316 clone showing interspersed blocks of HSR0 and telomere repeat DNA (TTTAGGG_n_). **(F)** The HuluTR180–270 clone showing a block of HSR0 repeats in one direction (blue) followed by blocks of inverted repeat polarity (red) in another.

We used dot plot and kmer analysis of long-read PacBio genomic sequence data to find tandem repeat candidates for new FISH probes. The two most frequent tandem repeat families found were the ~385 bp tandemly repeated sequence previously defined as the *Humulus*-specific subtelomeric repeat-1, HSR1 (Divashuk et al., 2014), and a new ~180 bp tandemly-repeated satellite DNA sequence designated HSR0 (*H. lupulus* Subterminal Repeat 0). As shown in Figure 2, the HSR0 repeat sequence arrays can span an entire sequence (Figure 2A), part of a PacBio sequence (Figure 2B), or arranged as blocks of inverted polarity within a single sequence (Figure 2F). The HSR0 repeats also showed an intriguing pattern, occasionally appearing adjacent to telomere repeat DNA (Figure 2D) or even interspersed with telomere repeats (Figure 2E). These observations suggest that HSR0 should make a good FISH probe that is predicted to be near telomeres. The HSR0 sequence is different than HSR1, and none of the HSR0 pac-bio clones examined included HSR1 repeats.

**Figure 3:**
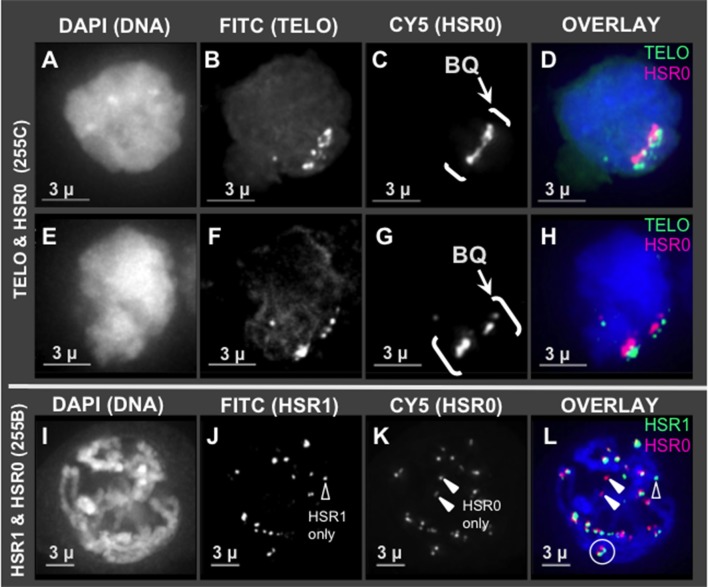
HSR0 co-localization with telomere and HSR1. Projections of 3D FISH datasets were produced as described in Figure 1 with an additional probe, HSR0. **(A–D,E–H)** Two hop nuclei from plant 255C at early prophase showing telomeres and HSR0 FISH signals clustered in one area on the nuclear periphery, the bouquet (bracketed, BQ). **(I–L)** One hop nucleus from plant 255B at middle-late prophase, homologous chromosomes paired in diplotene showing HSR0 and HSR1 co-localizing at the ends of chromosomes. **(J)** Showing HSR1 signal not co-localized (open arrowhead) and **(K)** showing two HSR0 signals not co-localized (closed arrowheads). **(L)** Circle denotes clear example of co-localization and polarity of the two FISH signals at the ends of chromosomes. Open and closed arrowheads show HSR1 and HSR0 signals, respectively, not co-localized. The length of the scale bars (3 microns) is indicated.

Given that HSR0 and telomere repeats occur together in clones, we predicted that HSR0 satellite DNA may be subtelomeric, and if so should stain bouquet and co-localize with HSR1. To test this, we carried out FISH using HSR0 together with either telomere (*n* = 21) or HSR1 (*n* = 13). Most of the HSR0 FISH signals did indeed colocalize with telomere FISH signals (Figures 3A–H). Most, but not all, of the HSR0 FISH signals also clearly co-localized with HSR1 FISH signals (Figures 3I–L). Despite the tendency for HSR0 and HSR1 to co-localize, we did find a few cases where only one was detected (arrowheads in Figures 2J,K). These solo signals from combined FISH probes could be useful for distinguishing otherwise similar chromosomes. Together, the molecular and cytogenetic analyses indicate that the 180 bp satellite repeat family HSR0 comprises a newly characterized and abundant subtelomeric tandem repeat sequence family. The HRS0 oligo FISH probe, ZERO-Y, represents a valuable new reagent for karyotyping and analysis of meiosis.

### Meiotic chromosome abnormalities are evident at mid-prophase, meiosis I, and II

**Figure 4:**
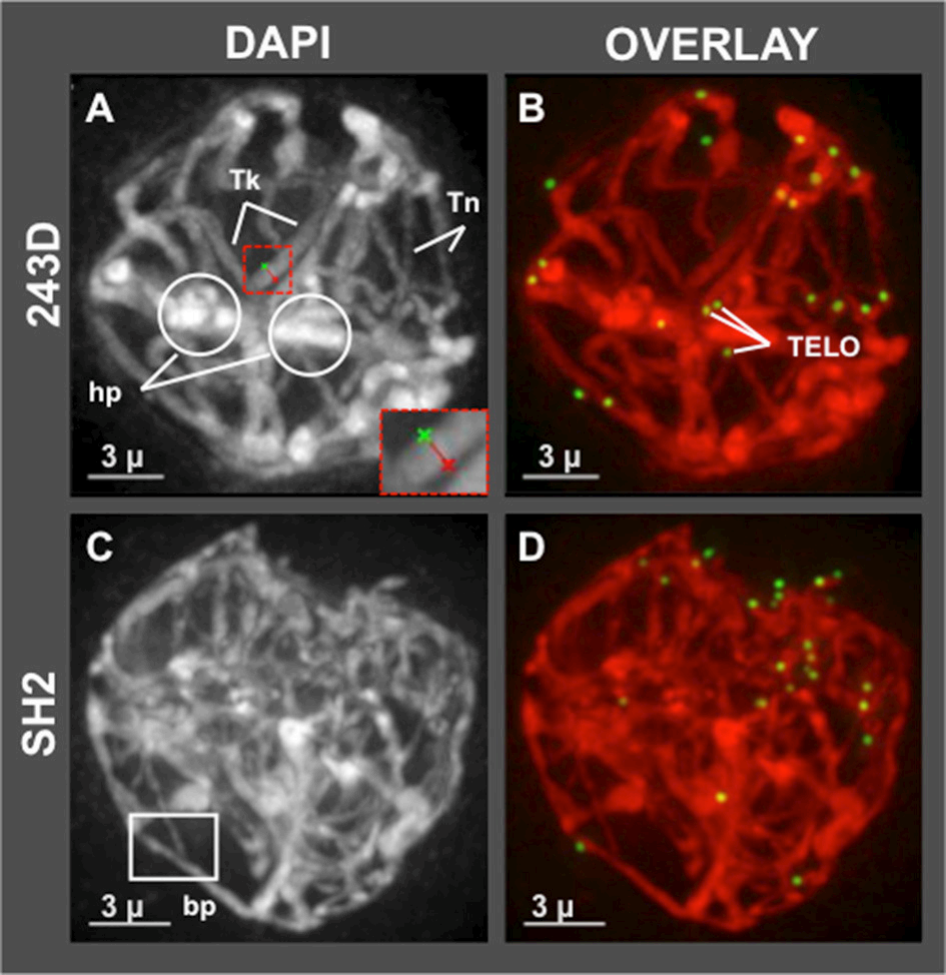
Hop pachytene nuclei with differential chromosome fiber thickness. Hop nuclei at pachytene showing distinct areas of non-uniform chromosome fibers. Fiber widths were measured at four locations per fiber and averaged, distinguishing thick (Tk) from thin (Tn) fibers. **(A,B)** Pachytene (middle prophase, post-bouquet) nucleus from plant 243D also shows heteropycnotic (circled, hp) regions. Red dashed box indicates an example of measurement tool for distance across fibers. **(C,D)** Middle prophase nucleus (early pachytene, post-bouquet) from wild-collected var. *neomexicanus*, plant SH2, shows a structure resembling a synapsis branch point (bp, white box). The length of the scale bars (3 microns) is indicated.

Having shown that hop has a normal bouquet (Figure 1), we wanted to examine in more detail the middle prophase/pachytene stage, when long thick fibers should appear and telomere distributions typically transition from clustered in early pachytene to dispersed in middle and late pachytene (Bass et al., 1997). We observed evidence of meiotic irregularities in pachytene, as shown in Figure 4. Specifically, all of the meiotic nuclei imaged at mid-prophase (*n* = 72 total from 7 different plants) showed conspicuous lack of uniformity of fiber appearance, a pattern resembling that of some meiotic mutants with disruption or loss of synchronous progression (Bass et al., 2003). For instance, in the example shown in Figure 4A, plant 243D and wild collected var. *neomexicanus* plant SH2, thick fiber (Tk) cross sections were measured and averaged 670–850 nm whereas thin (Tn) fibers averaged 320–460 nm. The fact that the telomeres are dispersed in this nucleus (Figure 4A) indicates that the cell has progressed beyond the bouquet stage which ends in early pachytene, at which point there should be no unpaired or unsynapsed chromosomes in a normal diploid cell. This bimodal fiber thickness is a conspicuous and invariant recurring phenotype in hop that appears to persist through all of pachytene. In addition to this conspicuous non-uniformity in fiber morphology, nuclei at this stage also showed heteropycnotic regions (hp, Figure 4A), possible entanglements, and presumed synapsis branch points (bp, Figure 4C).

**Figure 5:**
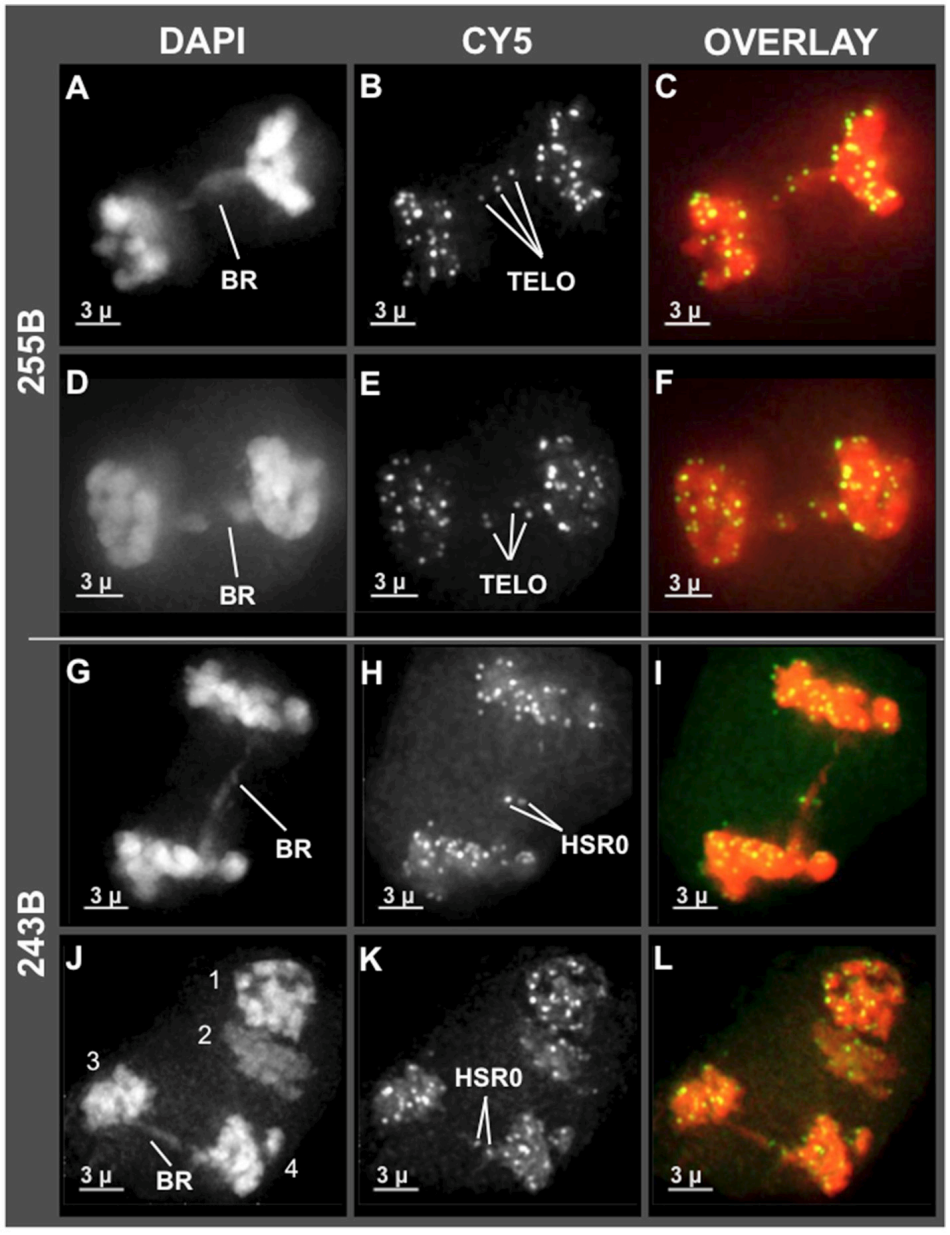
Chromosome bridges with interstitial telomere and HSR0 signals at meiosis I and II. Projections of 3D FISH datasets were produced as described in Figure 1. **(A–F)** Through-focus projections showing hop nuclei at anaphase I in plant 255B. The wavelength is indicated at top and the chromosome bridges (BR) and telomere FISH signals are (TELO) are indicated. **(G–L)** Through-focus projections of a hop nuclei at anaphase I **(G–I)** or anaphase II **(J–L)** from plant 243B. The chromosome bridges (BR) and HSR0 FISH signals (HSR0) are indicated. Telomere and HSR0 FISH signals can be observed on the bridges. The length of the scale bars (3 microns) is indicated.

Having observed unusual chromosome morphology in mid prophase (Figure 4), and non-disomic pairing in late prophase (Zhang et al., 2017), we next examined the stages immediately following meiotic prophase, the divisions of meiosis I and II. Some genera, such as *Oenothera* and *Clarkia*, can have translocation heterozygosity and meiotic chromosome complexes with surprisingly low segregation defects (Golczyk et al., 2014). It was therefore unclear if hop would deviate from the expectation of complete and balanced chromosome separation. To explore this question, we examined the first and second meiotic divisions for anomalies. We found that hop does exhibit anaphase bridges in both meiosis I and II as summarized in Figure 5. Bridges implicate dicentric chromosomes, which are indicative of inversions or pairing of rearranged chromosomes, and were observed in both Cascade (cross 243) and Apollo (cross 255) families (BR in Figures 5A,D,G,J). These bridges included interstitial FISH signals for telomeres (TELO, plant 243B, Figures 5B,E) or HSR0 (HSR0, plant 255B, Figures 5H,K). Hop appears to initially deviate from normal meiosis at some point in mid meiotic prophase, after the bouquet stage but prior to diakinesis just after homologous pairing and recombination typically occur (Zhang et al., 2017).

### Tetrad analysis with 5S rDNA FISH reveals both premeiotic aneuploidy and unbalanced segregation

We next set out to characterize the transmission of the 5S rDNA loci, which are discrete, euchromatic genic loci. The 5S rDNA FISH analyses also allowed us to survey segregation distortion in several contexts: within single plants, between progeny from a single cross, between different crosses, and between varieties of hop. For these experiments, the tetrad stage was selected because it is ideal for observing transmission genetics in a single generation, as the products of normal meiosis are expected to result in four daughter cells with equal segregation in a ratio of 1:1:1:1 per locus. Previously reported hop karyotyping (Karlov et al., 2003; Divashuk et al., 2011) showed that 5S rDNA loci reside on two chromosomes, one near the centromere of chromosome two and one in the telomeric region of chromosome five. In a somatic diploid nucleus stained by 5S rDNA FISH, signals therefore would appear as four distinct dots. During a normal Mendelian meiosis, signals would assort into the four daughter nuclei equally in a ratio of 2:2:2:2.

**Figure 6:**
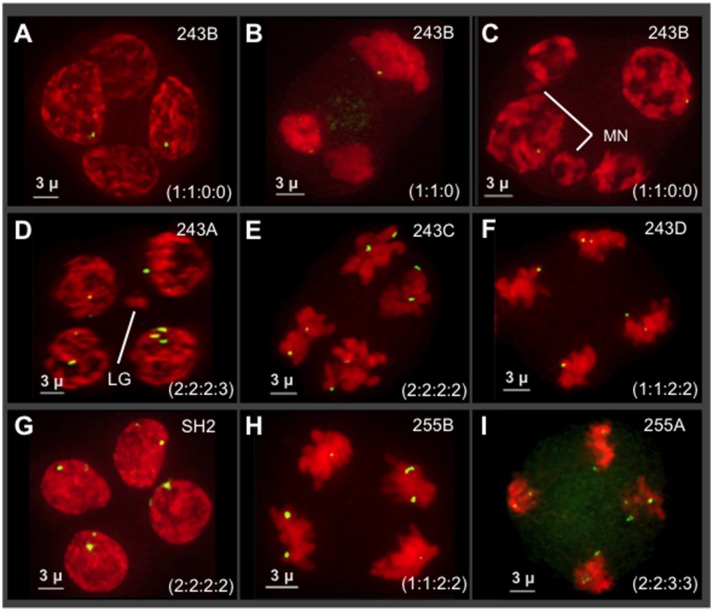
Survey of 5S rDNA FISH in tetrads (meiosis II) of seven different plants. Projections of 3D FISH datasets were produced as described in Figure 1 but with DAPI shown in red and 5S rDNA FISH, green. Hop tetrads are highly variable. **(A–F)** Four progeny of cross 243, in the Cascade family. **(A–C)** A single progeny of cross 243 from the plant designated 243B. **(D)** Plant 243A. **(E)** Plant 243C. **(F)** Plant 243D. **(G)** Plant SH2, a wild collected var. *neomexicanus* hop. **(H–I)** Two progeny of cross 255 from the Apollo family. **(H)** Plant 255B. **(I)** Plant 255A. The 5S rDNA ratios for each tetrad are listed in each panel. The occurrence of micronuclei (MN) and chromosome laggards (LG) are indicated in some of the panels. The length of the scale bars (3 microns) is indicated.

We examined seven different plants (*n* = 10–14 tetrads per plant) at the tetrad stage by 3D 5S rDNA FISH as summarized in Figure 6. The 5S rDNA FISH signals were also counted from multiple nuclei (*n* = 45–95 nuclei/plant) at different stages of meiosis in order to confirm the 5S rDNA constitution of plants and their progeny. We observed highly variable and extreme segregation defects as illustrated by tetrads from male progeny of cross 243 (Figure 6, top two rows). From images collected from a single plant (tetrads in Figures 6A–C), we found multiple types of non-Mendelian ratios including 1:1:0:0 and 1:1:0. These cells also sometimes included micronuclei or three nuclei, indicative of asynchronous division or whole nuclei non-disjunction (Figure 6B). Additional progeny of cross 243 showed even more segregation anomalies, including 5S rDNA tetrad ratios of 2:2:2:3 (Figure 6D) and 1:1:2:2 (Figure 6F) and chromosome micronuclei and laggards (MN in Figure 5C, LG in Figure 6D). Nuclei from two progeny of a separate cross, 255 in the Apollo family, confirmed that siblings from a single cross can show unbalanced 5S rDNA in tetrads (Figures 5H,I). Despite the frequency of abnormal 5S rDNA segregation patterns, we occasionally observed the normal, expected ratio of 2:2:2:2 in two cases, one from cross 243 (Figure 6E) and one from a wild-collected var. *neomexicanus* (Figure 6G). These observations show that the 5S rDNA ratios at the tetrad stage can vary between and within plants.

**Figure 7:**
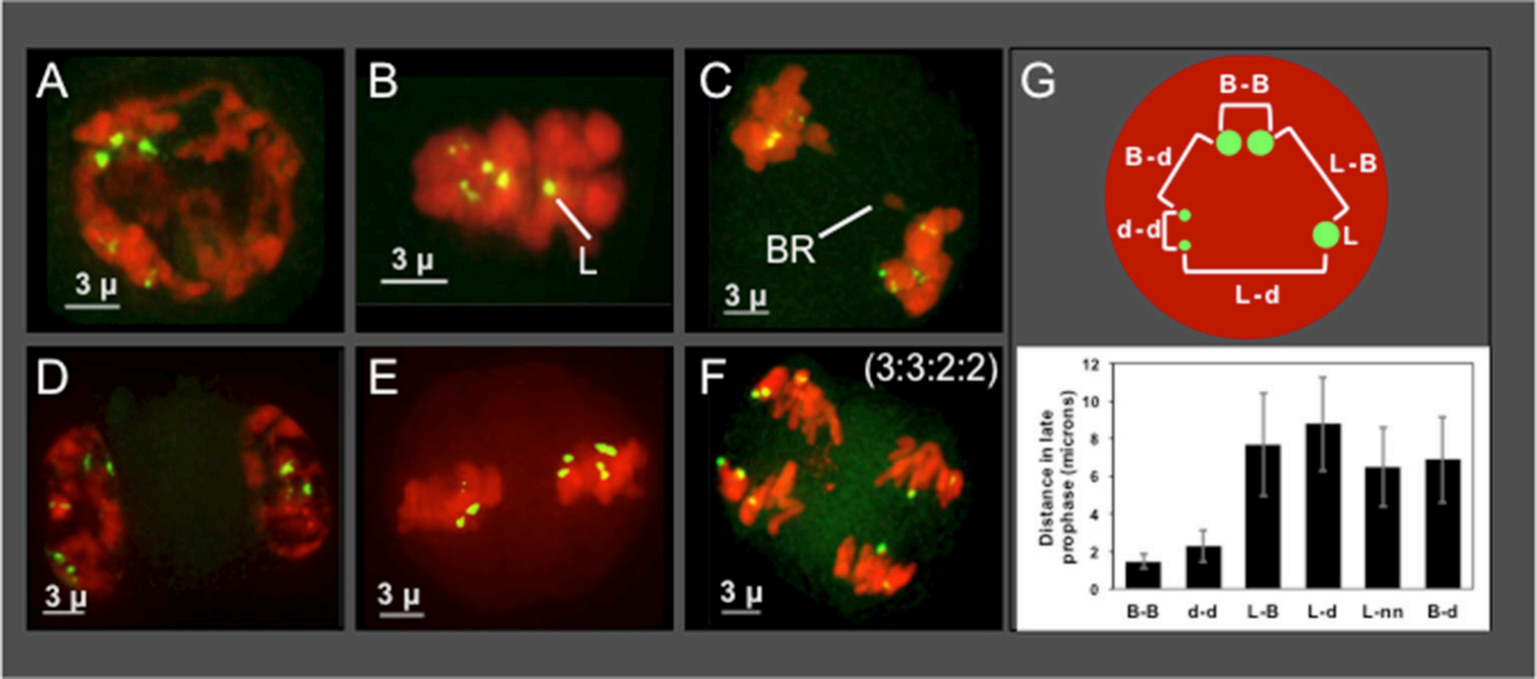
Plant 255A 5S rDNA FISH signal images through meiosis and distance mapping in late prophase. Projections of 3D FISH datasets were produced as described in Figure 1 but with DAPI shown in red and 5S rDNA FISH, green. **(A)** Early diplotene showing three bright 5S rDNA dots and two smaller and dimmer signals, referred to as the 5-locus pattern. **(B)** Metaphase I showing two dim double dots, representing homologous pairs of sister chromatids, and three bright dots, one of which is alone and designated the lone (L) signal. **(C)** Anaphase I showing a bridge (BR) and 5S rDNA FISH signals as 2 pairs (upper left) and 3 pairs (lower right). 5S rDNA FISH signals at **(D,E)** Telophase I **(D)** metaphase II **(E)**, and anaphase II **(F)** indicating the 5S rDNA FISH signal ratios (3:3:2:2) in the daughter nuclei. **(G)** Top: Schematic of pairwise distance measurements within a nucleus with FISH signals indicated (B, bright; d, dim; L, lone). Each distance mapped is represented by a bracket. Bottom: Graph of distance averages in microns. The length of the scale bars (3 microns) is indicated.

The progeny from Hopsteiner breeding cross 255 is of particular interest because it shows signs of meiotic problems from multiple lines of evidence, including diakinesis multi-valent complexes (Zhang et al., 2017), chromosome bridges (Figures 5A–F), and unbalanced 5S rDNA loci after meiosis II (Figures 6H,I), We were particularly interested in plant 255A because it showed a recurring and obvious pattern of 2:2:3:3 segregation in meiotic daughter cells (Figure 6I), which is distinct from both normally expected ratios and from those of its sibling 255B (Figure 6H). Previously reported European karyotypes (Karlov et al., 2003; Divashuk et al., 2014) have two unlinked 5S rDNA loci. As summarized in Figure 7, we frequently observed three bright and two dim 5S rDNA FISH loci per nucleus during meiotic prophase (Figure 7A) in cells (*n* = 61) from 255A. This 5-locus pattern is also seen at metaphase I (Figure 7B), where one of the three bright dots appears to be alone, and unpaired (labeled “L” in Figure 7B). After meiotic prophase, the FISH signals for each locus often appear as double dots likely reflecting slight spatial separation of sister chromatid signals. The late anaphase I nucleus (Figure 7C) shows a chromosome bridge and an unbalanced distribution of 5S rDNA signals (Figure 7C). At telophase I, metaphase II, and anaphase II we find additional evidence for the 5-locus pattern (Figures 7D–F).

Regarding the nature of the three bright 5S rDNA loci, which reflect some type of aneuploidy, segmental or chromosomal, we wanted to distinguish between two explanatory scenarios—trisomy with 3 homologous loci or disomy plus a single 5S rDNA locus surrounded by non-homologous segments. If trisomic, the three bright loci should co-localize during pairing, but if disomic plus monosomic then the monosomic locus should show no preferential co-localization with the other bright dots. We measured pairwise distances in 3D space for the five 5S rDNA signals in late meiotic prophase nuclei (Figure 7G). The lone bright signal was found to be no closer to the other bright signals than to the dim signals, a pattern consistent with one disomic pair and one monosomic, unlinked locus. The chromosome bearing the lone 5S rDNA signal in nuclei from plant 255A has nonetheless been observed to pair with one or more other chromosomes (Figure S1, and spinning projection movie File S1), suggesting that the chromosome bearing the “lone” signal may share homology with other chromosomes. Notably, this plant illustrates but one of several types of deviation from the expected 5S rDNA pattern of 2:2:2:2. Other plants have their own characteristic 5S rDNA aneuploidy, as illustrated by the 3 tetrads from plant 243B (Figures 6A–C).

### Pedigree and FACS data show alternating DNA content over multiple generations

**Figure 8:**
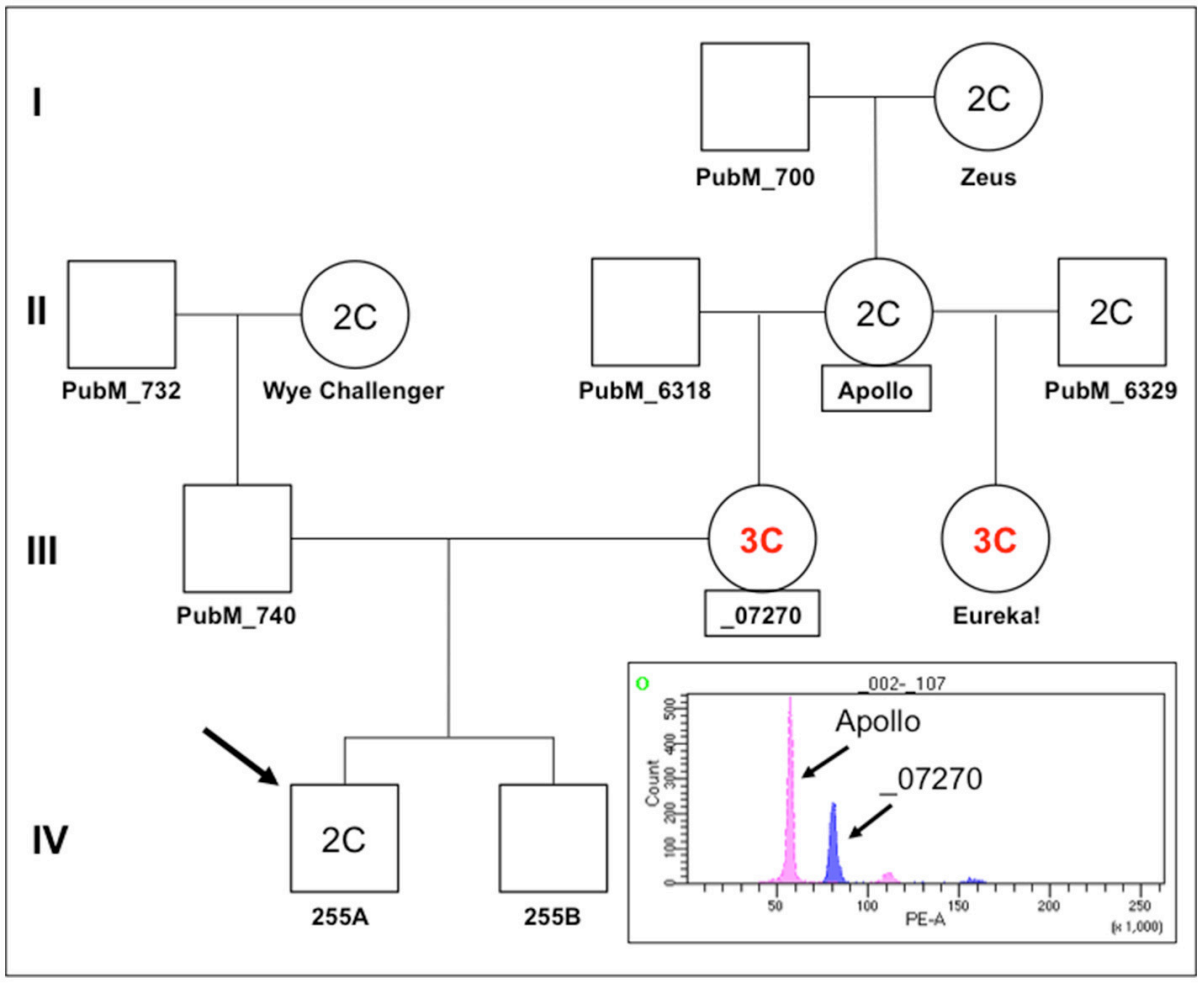
Pedigree of Apollo family and FACS. A partial pedigree of hops relevant to this study was constructed and annotated. Fluorescence-activated cell sorting was carried out for several plants in the Apollo family in relation to plant 255A (IV,1, the proband/arrow). Where determined by FACS, the DNA content is indicated as either 2C or 3C. The histogram showing fluorescence intensity peaks for nuclei from Apollo around 56 PE-A (2C) and for nuclei from _07270 around 80 PE-A (3C). Smaller fluorescence peaks at about double each main peak can also be observed at ~110 and ~160, indicative of nuclei that have likely undergone one round of endoreduplication.

In order to investigate the nature of segregation distortion of hop in a broader context, we examined the hop family tree in relation to cross 255 as summarized in Figure 8. In this pedigree, plant 255A is the proband and it has a DNA content designated 2C, similar to that of a reference diploid, Apollo (Figure 8, FACS inset). We noted an unexpected and intriguing DNA content pattern whereby the first two generations (I and II) have plants with 2C genome content but the third (III) has two female progeny of Apollo with 3C genome content (_07270 and Eureka!), followed by a fourth generation (IV) with 2C genome content (255A). Notably, two female progeny of Apollo, with two different male parents, both have 3C genome content. The 3C content of _07270 provides a possible explanation for why some of its progeny appear aneuploid (e.g., Figure 7, for 255A). Histograms for genome content of all other notated plants in the pedigree are shown in Figure S2.

## Discussion

The hop breeding industry has long grappled with segregation distortion and non-Mendelian inheritance, but the causal mechanisms of these problems remain largely unexplained. Here, we used 3D molecular cytology to ask at what point does hop first appear to deviate from the normal processes of meiosis. We found that early meiotic prophase appears normal, with a diagnostic telomere bouquet signifying proper commencement of the homology search process. The first clear signs of meiotic chromosome irregularities in hops appear in middle prophase, when the effects of aneuploidy should become apparent and readily detectable via 3D imaging. Specifically, we found a mixture of thick and thin pachytene fibers, interpreted as paired and unpaired, respectively, similar to those seen with the meiosis-specific maize mutant *desynaptic1* (Golubovskaya et al., 1997; Bass et al., 2003). We have considered several possible explanations for such non-uniform fiber appearance at mid-prophase, including differential progression of chromatin condensation or axial contraction or, more likely, the persistence of non-synapsed chromosome regions.

Non-disomic pairing in hop has been reported for chromosome complexes that include the XY sex bivalents (Sinotô, 1929) and those involving only autosomes (Zhang et al., 2017). Polyploidy, aneuploidy, paracentric inversions, and translocation heterozygosity can all lead to chromosomes with extra or missing centromeres, resulting in anaphase bridges or chromosome laggards. Although we do not know the extent to which each of these occur in hop, we found that nearly half of the cells examined in this study displayed bridges at anaphase I and II. These bridges should be prone to breakage, which could lead to the breakage-fusion-bridge (BFB) cycle as described by Barbara McClintock in maize (McClintock, 1939, 1941). She observed that radiation-induced chromosome breakage could initiate a BFB cycle in which broken ends can fuse and recreate more bridge-producing break-inducing dicentric chromosomes. BFB-generated acentric fragments can also become laggards and micronuclei which are not transmitted to progeny, and thus contribute to loss of genetic material and partial aneuploidy. We observed all the hallmarks of the BFB, including bridges, laggards, and micronuclei. Consequently, the BFB cycle can create a cascade of downstream chromosome structural variants, which in turn creates new problems for the next generation (Zheng et al., 1999; Han et al., 2006, 2009; Liu et al., 2015). We propose that the persistence of unpaired regions well into pachytene and the generation of anaphase bridges are the natural consequence of structural heterozygosity or aneuploidy, and through a BFB process could generate even more genomic structural diversity.

To untangle some of the complexities of hop meiosis, we FISH-stained the highly conserved 5S rDNA sequences which conveniently mark discrete, euchromatic genic loci. This study clearly shows that the number of 5S rDNA loci can be different in individual plants, even among siblings with the same parents. This premeiotic 5S rDNA aneuploidy has not been shown before, but is consistent with the emerging picture of structural genomic diversity. From a practical point of view, the 5S rDNA oligo FISH probes provide one efficient method to cytotype somatic seedling tissue in progeny to ascertain the severity of aneuploidy (an odd number of FISH signals) from a given cross.

Among the discoveries reported in this study is the identification and characterization of a new subtelomeric satellite DNA designated here as HSR0. HSR0 has a high %A+T content (63% for clone HuluTR180-120, GenBank MH188533) and is similar to the previously reported sub-terminal tandem repeat family HSR1 (Divashuk et al., 2011). However, unlike HSR1, we found several cases where the HSR0 repeats were in the same PacBio clone as telomere repeat sequences from Apollo DNA. This suggests that HSR0 may be the most distal non-telomeric repeat sequence. The interdigitation of telomere repeats with HSR0 is a curious feature that may implicate HSR0 in telomeric recombination, although multiple dispersed repeat clusters are not normally recombinagenic. Tandem repeats will be useful as a FISH probes for karyotyping different varieties of hop, for facilitating genome assemblies, and for identifying areas of aneuploidy and monitoring homologous pairing and segregation during meiosis.

Polyploidization and variable cytotypes are common in plants (Kolár et al., 2017) and can be advantageous due to gene redundancy and optimization of heterozygosity (Comai, 2005). Hop has been shown to tolerate variable ploidy, including triploidy, which can produce desirable traits such as seedlessness (Haunold, 1970, 1971, 1974; Beatson and Brewer, 1994; Beatson et al., 2003). In a study of crosses between triploid and diploid plants, a series of aneuploid plants with chromosome numbers between 20 and 55 were observed (Haunold, 1970). In a more recent study, triploid plants, which are generally expected to be sterile, were open pollinated with other triploid plants, and FACS analysis of progeny revealed a range of polyploidy ranging from haploid to tetraploid (Beatson et al., 2003). These results were similar to Haunold's, noting that FACS was unable to resolve minor aneuploidies such as monosomy (2n−1) or trisomy (2n+1). Here, we report that plants in the Apollo family can have variable DNA content, which predicts meiotic anomalies. In considering how two parents with 2C DNA content can produce 3C progeny (Figure 8, Eureka from Apollo x PubM_6329), we occasionally observed cases where meiosis II whole genome non-disjunction seems to have occurred in a male (e.g., Figure 6B). If such a non-reduced nucleus with two sets of chromosomes fertilized a normal haploid egg nucleus, a triploid zygote could result, providing one possible mechanism for a shift in DNA content. It will be interesting to explore whether or not there might be a natural genetic contribution to these DNA content switches.

In this study, we have used 3D imaging to investigate meiotic chromosome behavior at stages that are difficult to study using conventional squash or spread techniques. We found that hop has a canonical bouquet, a structure that occurs in zygotene and early pachytene. In contrast, following the bouquet stage, hop chromosomes begin to show conspicuous and dramatic deviation from the normal progression of chromosome morphology and behavior. From these data we conclude that pairing and synapsis irregularities commence in mid-prophase and later manifest in chromosome bridges, breaks, and non-disomic assortment with varied degrees of complexity. In addition, the occurrence of 3C DNA content plants in the hop lineages may further contribute to genomic instability. Taken together, these findings reveal that there may be multiple and complex mixtures of contributing factors to the segregation distortion of hop, with the possibility that the majority of transmission anomalies are associated with domestication and breeding. However, very few native North American wild accessions have been cytologically analyzed. It will therefore be important to systematically investigate meiosis in multiple truly wild and native North American populations. Developing a more comprehensive understanding of transmission genetics in both wild and cultivated hop species will facilitate and accelerate efforts to meet the growing demands for new hop varieties in all of its associated industries.

## Author contributions

KE, HB, RJ, and PM, collected and processed samples for microscopy. NP and DZ collected and analyzed genomic data. KE collected and analyzed 3D image data. KE, HB, LL, and JA characterized and tested tandem repeat FISH probes. RJ and PM obtained FACS data and NP helped develop the pedigree analysis. KE and HB produced the figures, analyzed the cytogenetics and were primary in the design and interpretation of the results.

### Conflict of interest statement

The authors declare that the research was conducted in the absence of any commercial or financial relationships that could be construed as a potential conflict of interest.

## Supplementary material

Figure S1 Plant 255A highlighting the lone 5S rDNA-bearing chromosome at diakinesis. Projections of 3D FISH datasets were produced as described in Figure 1. Two nuclei from plant 255A at diplotene-diakinesis **(A,D)** show pairs (BB, dd) of 5S rDNA signals (green) and the lone, unpaired signal (L) in a whole-nucleus through-focus projection. Chromosomes (DAPI, red) bearing the lone signals were cropped out of the nucleus and shown from two angles **(B,C,E,F)**. The nucleus in panel D is also shown as spinning projections movie (File S1).

Figure S2 FACS data for DNA content of selected hops. PE-A fluorescence intensity histograms for leaf-tissue nuclei are shown for plants from the pedigree shown in Figure 8. Plants called with 2C DNA content (peaks around 58 PE-A, **(A,B,C,E)** or with 3C DNA content (peak around 80 PE-A, **(D)** are shown along with the names of the individual plants.

File S1 Spinning projection movie of 5S rDNA signals at late prophase from plant 255A. Quicktime movie showing maximum intensity projections of 3D dataset from plant 255A (KAE_CC5a_D3D_CAC_VOL_XY). A single diakinesis nucleus DAPI (red) and 5S rDNA FISH signals (green) are shown. This nucleus is the same as that shown in Figure S1D.

## References

[B1] Albert P. S. Gao Z. Danilova T. V. Birchler J. A. (2010). Diversity of chromosomal karyotypes in maize and its relatives. Cytogenet. Genome Res. 129, 6–16. 10.1159/000314342 20551613

[B2] Alexandrov O. S. Divashuk M. G. Yakovin N. A. Karlov G. I. (2012). Sex chromosome differentiation in *Humulus japonicus* Siebold & Zuccarini, 1846 (Cannabaceae) revealed by fluorescence *in situ* hybridization of subtelomeric repeat. Comp. Cytogenet. 6, 239–247. 10.3897/compcytogen.v6i3.3261 24260665 PMC3833800

[B3] Bass H. W. (2003). Telomere dynamics unique to meiotic prophase: formation and significance of the bouquet. Cell. Mol. Life Sci. 60, 2319–2324. 10.1007/s00018-003-3312-4 14625678 PMC11138934

[B4] Bass H. W. Bordoli S. J. Foss E. M. (2003). The desynaptic (dy) and desynaptic1 (dsy1) mutations in maize (*Zea mays* L.) cause distinct telomere-misplacement phenotypes during meiotic prophase. J. Exp. Bot. 54, 39–46. 10.1093/jxb/erg032 12456753

[B5] Bass H. W. Marshall W. F. Sedat J. W. Agard D. A. Cande W. Z. (1997). Telomeres cluster *de novo* before the initiation of synapsis: a three-dimensional spatial analysis of telomere positions before and during meiotic prophase. J. Cell Biol. 137, 5–18. 10.1083/jcb.137.1.5 9105032 PMC2139864

[B6] BassH. W.Riera-LizarazuO.AnanievE. V.BordoliS. J.RinesH. W.PhillipsR. L.. (2000). Evidence for the coincident initiation of homolog pairing and synapsis during the telomere-clustering (bouquet) stage of meiotic prophase. J. Cell Sci. 113 (Pt 6), 1033–1042. 1068315110.1242/jcs.113.6.1033

[B7] Beatson R. A. Brewer V. R. (1994). Regional trial evaluation and cultivar selection of triploid hop hybrids. N. Z. J. Crop Hortic. Sci. 22, 1–6. 10.1080/01140671.1994.9513799

[B8] Beatson R. A. Ferguson A. R. Weir I. E. Graham L. T. Ansell K. A. Ding H. (2003). Flow cytometric identification of sexually derived polyploids in hop (*Humulus lupulus* L.) and their use in hop breeding. Euphytica 134, 189–194. 10.1023/B:EUPH.0000003882.23615.c5

[B9] Comai L. (2005). The advantages and disadvantages of being polyploid. Nat. Rev. Genet. 6, 836–846. 10.1038/nrg1711 16304599

[B10] Divashuk M. G. Alexandrov O. S. Kroupin P. Y. Karlov G. I. (2011). Molecular cytogenetic mapping of *Humulus lupulus* sex chromosomes. Cytogenet. Genome Res. 134, 213–219. 10.1159/000328831 21709414

[B11] Divashuk M. G. Alexandrov O. S. Razumova O. V. Kirov I. V. Karlov G. I. (2014). Molecular cytogenetic characterization of the dioecious Cannabis sativa with an XY chromosome sex determination system. PLoS ONE 9:e85118. 10.1371/journal.pone.0085118 24465491 PMC3897423

[B12] Golczyk H. Massouh A. Greiner S. (2014). Translocations of chromosome end-segments and facultative heterochromatin promote meiotic ring formation in evening primroses. Plant Cell 26, 1280–1293. 10.1105/tpc.114.122655 24681616 PMC4001384

[B13] Golczyk H. Musiał K. Rauwolf U. Meurer J. Herrmann R. G. Greiner S. (2008). Meiotic events in Oenothera - a non-standard pattern of chromosome behaviour. Genome 51, 952–958. 10.1139/G08-081 18956028

[B14] Golubovskaya I. N. Grebennikova Z. K. Auger D. L. Sheridan W. F. (1997). The maize *desynaptic1* mutation disrupts meiotic chromosome synapsis. Dev. Genet. 21, 146–159. 10.1002/(SICI)1520-6408(1997)21:2<146::AID-DVG4>3.0.CO;2-7

[B15] Han F. Gao Z. Birchler J. A. (2009). Reactivation of an inactive centromere reveals epigenetic and structural components for centromere specification in maize. Plant Cell 21, 1929–1939. 10.1105/tpc.109.066662 19602622 PMC2729603

[B16] Han F. Lamb J. C. Birchler J. A. (2006). High frequency of centromere inactivation resulting in stable dicentric chromosomes of maize. Proc. Natl. Acad. Sci. U.S.A. 103, 3238–3243. 10.1073/pnas.0509650103 16492777 PMC1413895

[B17] Haunold A. (1970). Fertility studies and cytological analysis of the progeny of a triploid × diploid cross in hop, *Humulus lupulus* L. Can. J. Genet. Cytol. 12, 582–588. 10.1139/g70-077

[B18] Haunold A. (1971). Cytology, sex expression, and growth of a tetraploid × diploid cross in hop (*Humulus lupulus* L.). Crop Sci. 11, 868–871. 10.2135/cropsci1971.0011183X001100060031x

[B19] Haunold A. (1974). Meiotic chromosome behavior and pollen fertility of a triploid hop 1. Crop Sci. 14, 849–852. 10.2135/cropsci1974.0011183X001400060022x

[B20] Haunold A. (1991). Cytology and cytogenetics of hops, in Chromosome Engineering in Plants: Genetics, Breeding, Evolution, eds Gupta P. K. Tsuchiya T.(Amsterdam), 551–563.

[B21] Howe E. S. Murphy S. P. Bass H. W. (2013). Three-dimensional acrylamide fluorescence *in situ* hybridization for plant cells. Methods Mol. Biol. 990, 53–66. 10.1007/978-1-62703-333-6_6 23559202

[B22] Jacobsen P. (1957). The sex chromosomes in *Humulus*. Hereditas 43, 357–370. 10.1111/j.1601-5223.1957.tb03444.x

[B23] Jakse J. Stajner N. Kozjak P. Cerenak A. Javornik B. (2008). Trinucleotide microsatellite repeat is tightly linked to male sex in hop (*Humulus lupulus* L.). Mol. Breed. 21, 139–148. 10.1007/s11032-007-9114-x

[B24] Jakse J. Stajner N. Luthar Z. Jeltsch J.-M. Javornik B. (2011). Development of transcript-associated microsatellite markers for diversity and linkage mapping studies in hop (*Humulus lupulus* L.). Mol. Breed. 28, 227–239. 10.1007/s11032-010-9476-3

[B25] John B. (1990). Meiosis. Cambridge, UK: Cambridge University Press.

[B26] Karlov G. I. Danilova T. V. Horlemann C. Weber G. (2003). Molecular cytogenetics in hop (*Humulus lupulus* L.) and identification of sex chromosomes by DAPI-banding. Euphytica 132, 185–190. 10.1023/A:1024646818324

[B27] Kolár F. Certner M. Suda J. Schönswetter P. Husband B. C. (2017). Mixed-ploidy species: progress and opportunities in polyploid research. Trends Plant Sci. 22, 1041–1055. 10.1016/j.tplants.2017.09.011 29054346

[B28] Liu Y. Su H. Pang J. Gao Z. Wang X. J. Birchler J. A.. (2015). Sequential *de novo* centromere formation and inactivation on a chromosomal fragment in maize. Proc. Natl. Acad. Sci. U.S.A. 112, E1263–E1271. 10.1073/pnas.1418248112 25733907 PMC4371999

[B29] McAdam E. L. Freeman J. S. Whittock S. P. Buck E. J. Jakse J. Cerenak A.. (2013). Quantitative trait loci in hop (*Humulus lupulus* L.) reveal complex genetic architecture underlying variation in sex, yield and cone chemistry. BMC Genomics 14:360. 10.1186/1471-2164-14-360 23718194 PMC3680207

[B30] McAdam E. L. Vaillancourt R. E. Koutoulis A. Whittock S. P. (2014). Quantitative genetic parameters for yield, plant growth and cone chemical traits in hop (*Humulus lupulus* L.). BMC Genet. 15:22. 10.1186/1471-2156-15-22 24524684 PMC3927220

[B31] McClintock B. (1939). The behavior in successive nuclear divisions of a chromosome broken at meiosis. Proc. Natl. Acad. Sci. U.S.A. 25, 405–416. 10.1073/pnas.25.8.405 16577924 PMC1077932

[B32] McClintock B. (1941). The stability of broken ends of chromosomes in zea mays. Genetics 26, 234–282. 17247004 10.1093/genetics/26.2.234 PMC1209127

[B33] Murphy S. P. Bass H. W. (2012). Genetics and cytology of meiotic chromosome behavior in plants, in Plant Cytogenetics: Genome Structure and Chromosome Function, eds Bass H. W. Birchler J. A. (New York, NY: Springer New York), 193–229.

[B34] Neve R. A. (1958). Sex chromosomes in the hop *Humulus lupulus*. Nature 181, 1084–1085. 10.1038/1811084b0

[B35] Neve R. A. (ed.). (1991). Hops. London, UK: Chapman and Hall.

[B36] Ohtsubo Y. Ikeda-Ohtsubo W. Nagata Y. Tsuda M. (2008). GenomeMatcher: a graphical user interface for DNA sequence comparison. BMC Bioinformatics 9:376. 10.1186/1471-2105-9-376 18793444 PMC2553346

[B37] Pellicer J. Leitch I. J. (2014). The application of flow cytometry for estimating genome size and ploidy level in plants. Methods Mol. Biol. 1115, 279–307. 10.1007/978-1-62703-767-9_14 24415480

[B38] Reeves P. A. Richards C. M. (2011). Species delimitation under the general lineage concept: an empirical example using wild North American hops (Cannabaceae: *Humulus lupulus*). Syst. Biol. 60, 45–59. 10.1093/sysbio/syq056 21088008

[B39] Roborgh R. H. J. (1969). The production of seedless varieties of hop (*Humulus lupulus*) with colchicine. N. Z. J. Agri. Res. 12, 256–259. 10.1080/00288233.1969.10427094

[B40] Scherthan H. (2007). Telomere attachment and clustering during meiosis. Cell. Mol. Life Sci. 64, 117–124. 10.1007/s00018-006-6463-2 17219025 PMC11136177

[B41] Seefelder S. Ehrmaier H. Schweizer G. Seigner E. (2000). Male and female genetic linkage map of hops, *Humulus lupulus*. Plant Breed. 119, 249–255. 10.1046/j.1439-0523.2000.00469.x

[B42] Shephard H. L. Parker J. S. Darby P. (2000). Sexual development and sex chromosomes in hop. New Phytol. 148, 397–411. 10.1046/j.1469-8137.2000.00771.x/full 33863027

[B43] Sinotô Y. (1929). On the tetrapartite chromosome in *Humulus lupulus*. Proc. Imp. Acad. 5, 46–47. 10.2183/pjab1912.5.46

[B44] Small E. (1980). The relationships of hop cultivars and wild variants of *Humulus lupulus*. Can. J. Bot. 58, 676–686. 10.1139/b80-086

[B45] Stebbins G. L. (1935). Chromosome structure and the mechanism of meiosis in plants. Am. Nat. 69:81.

[B46] Szymanski M. Barciszewska M. Z. Erdmann V. A. Barciszewski J. (2002). 5S Ribosomal RNA database. Nucleic Acids Res. 30, 176–178. 10.1093/nar/30.1.176 11752286 PMC99124

[B47] Winge Ö. (1929). On the nature of the sex chromosomes in *Humulus*. Hereditas 12, 53–63. 10.1111/j.1601-5223.1929.tb02497.x

[B48] Zhang D. Easterling K. A. Pitra N. J. Coles M. C. Buckler E. S. Bass H. W.. (2017). Non-mendelian single-nucleotide polymorphism inheritance and atypical meiotic configurations are prevalent in hop. Plant Genome 10:14. 10.3835/plantgenome2017.04.0032 29293819

[B49] Zheng Y. Z. Roseman R. R. Carlson W. R. (1999). Time course study of the chromosome-type breakage-fusion-bridge cycle in maize. Genetics 153, 1435–1444. 10545471 10.1093/genetics/153.3.1435 PMC1460833

