# Supplementary figures and images for: 3D Molecular Cytology of Hop (Humulus lupulus) Meiotic Chromosomes Reveals Non-disomic Pairing and Segregation, Aneuploidy, and Genomic Structural Variation
Source: Front Plant Sci. 2018 Nov 1;9:1501. doi: 10.3389/fpls.2018.01501 (PMC6221928; doi:10.3389/fpls.2018.01501)

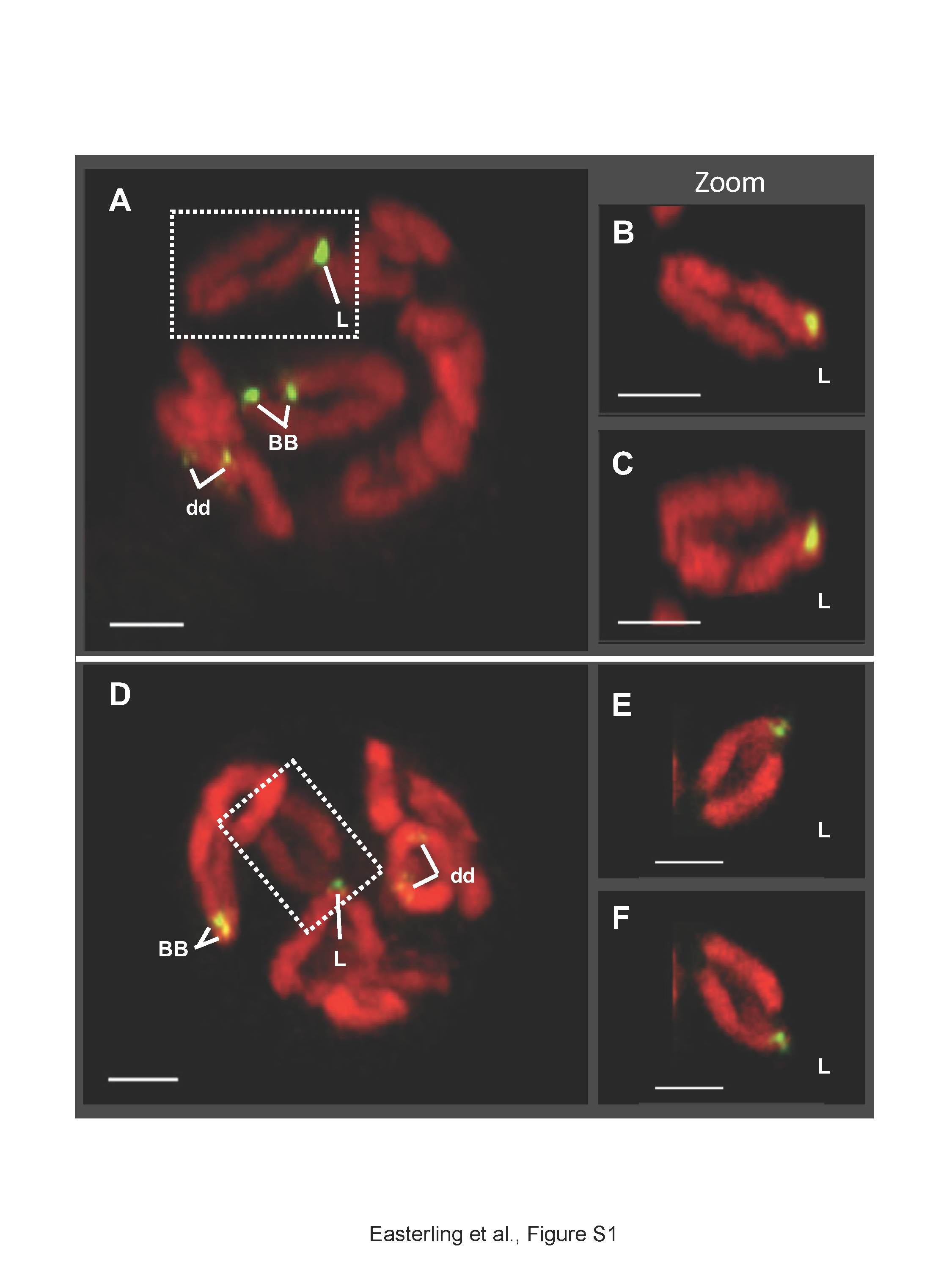

Supplement: Figure S1 — Plant 255A highlighting the lone 5S rDNA-bearing chromosome at diakinesis. Projections of 3D FISH datasets were produced as described in Figure 1. Two nuclei from plant 255A at diplotene-diakinesis (A,D) show pairs (BB, dd) of 5S rDNA signals (green) and the lone, unpaired signal (L) in a whole-nucleus through-focus projection. Chromosomes (DAPI, red) bearing the lone signals were cropped out of the nucleus and shown from two angles (B,C,E,F). The nucleus in panel D is also shown as spinning projections movie (File S1). [file Image_1.TIFF]

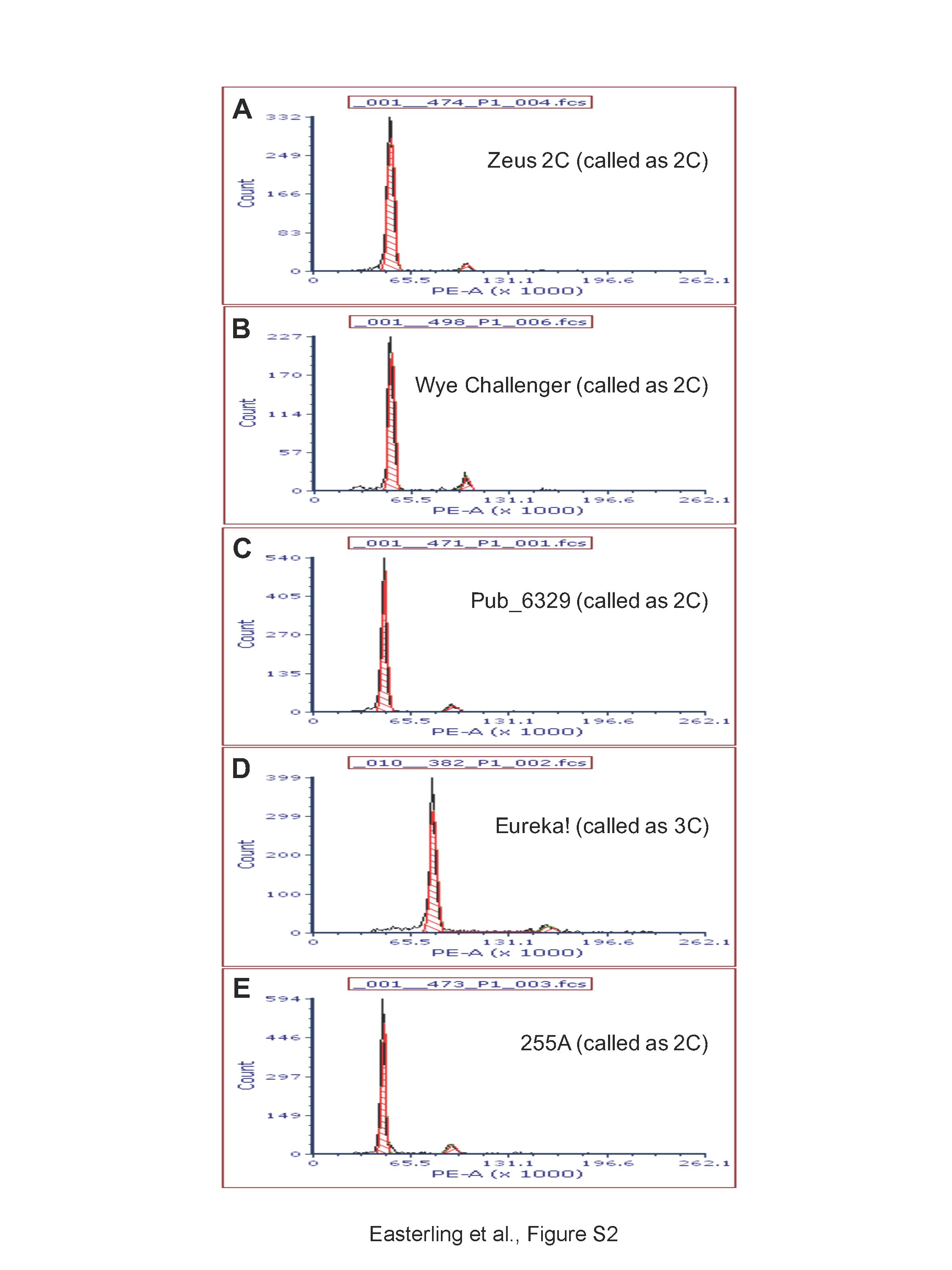

Supplement: Figure S2 — FACS data for DNA content of selected hops. PE-A fluorescence intensity histograms for leaf-tissue nuclei are shown for plants from the pedigree shown in Figure 8. Plants called with 2C DNA content (peaks around 58 PE-A, (A,B,C,E) or with 3C DNA content (peak around 80 PE-A, (D) are shown along with the names of the individual plants. [file Image_2.TIFF]
